# A standardized protocol for anal fistula induction in a rat model

**DOI:** 10.1590/acb406125

**Published:** 2025-08-18

**Authors:** Paulo Cesar de Castro, Jenif Braga de Souza, Ruy Garcia Marques

**Affiliations:** 1 Universidade do Estado do Rio de Janeiro – Faculty of Medical Sciences – Department of General Surgery –Rio de Janeiro (RJ) – Brazil.

**Keywords:** Rats, Wistar, Rectal Fistula, Models, Animal

## Abstract

**Purpose:**

To evaluate an experimental model of anal fistulas in rats, analyze the primary challenges associated with this model, and identify its key advantages.

**Methods:**

Twenty-six male Wistar rats underwent surgical induction of two anal fistulas each, using a metal seton. The setons remained in place for 30 days, after which they were removed. Animals were divided in two groups (n = 13 per group). Group 1 was euthanized two weeks post-seton removal, and Group 2 in four weeks post-seton removal. Two fistula tissue samples were collected from each animal for histopathological analysis.

**Results:**

All 26 animals tolerated the procedure well, with an average weight gain of 64.3 grams in 30 days. There was no animal loss during the experiment, but eight setons were lost due to poor fixation. One animal lost both setons and had to be excluded from the experiment, resulting in a 96% success rate. All 44 remaining fistulas could be identified by observing the scar on the external orifice, two and four weeks after the removal of the metal wire.

**Conclusion:**

The two groups showed no differences. The experimental model in this work proved to be quite effective, economically viable and easy to reproduce.

## Introduction

Among diseases that affect the perianal region, anal fistulas have a high incidence. The prevalence of anal fistulas can reach 5.6 cases per 100,000 women and 12.3 cases per 100,000 men^1^. Surgical intervention is the primary treatment for this condition. The most significant risk associated with this treatment approach is the potential impairment of anal sphincter function. Two classic approaches to treat anal fistulas include seton placement, documented by Hippocrates, and fistulotomy, described since the time of Galen^2^. While both techniques remain widely utilized, they have limitations. The techniques may not effectively address the diverse anatomical presentations of anal fistulas, and most importantly, they are associated with a significant risk of sphincter damage. Alternative treatment modalities have been proposed, namely, occlusion of the internal orifice of the fistula by advancing a myomucosal flap^3^; intersphincteric ligation of the fistula tract^4^; occlusion of the fistula tract with glue^5^, among others. All these techniques aim to preserve sphincter function as much as possible. However, we still do not have a gold standard alternative that both results in effective healing and carries minimal risk of sphincter impairment, especially for the treatment of complex anal fistulas.

The development of techniques for the treatment of anal fistulas requires study, historical knowledge, and innovation, especially when novel approaches are sought. Thus, the initial and experimental investigation of new therapies for the treatment of anal fistulas in humans may present risks and have undesirable implications for good scientific practice. Consequently, the investigation and development of novel therapeutic alternatives depend on the utilization of experimental animal models that are readily reproducible, cost-effective, and ethically sound.

Several animal models have been previously utilized in research, with small mammals such as rats^6^–^9^ or rabbits^10^,^11^ serving as common experimental models. Other models involve larger animals, such as dogs^12^. Indeed, this animal already develops its own perianal disease, namely canine anal furunculosis, which is similar to the anal fistula of cryptoglandular origin that is observed in humans^13^. Different models involving pigs have been suggested^14^,^15^. The difficulties in handling and housing these larger animals make their use in larger-scale experiments difficult and expensive. Models based on smaller animals, such as rats, have several advantages, and the purpose of this study was to illustrate the use, advantages, and disadvantages of these models.

The, the objective of this article was to demonstrate an experimental model for the study of induced anal fistulas in rats, reviewing its main technical difficulties.

## Methods

The work was conducted as an experimental study that was developed at the Experimental Surgery Laboratory of the Faculty of Medical Sciences at Universidade do Estado do Rio de Janeiro (UERJ). This study was approved by the Ethics Committee for the Care and Use of Experimental Animals, Roberto Alcantara Gomes Institute of Biology, under the number 043/2023.

To conduct this experiment, 26 male Wistar rats (*Rattus norvegicus*), with the average weight of 311 grams, were used. The animals were obtained from the vivarium of the Experimental Surgery Laboratory and were housed there, in cages with three animals each, exposed to 12-hour periods of daylight. They received water and appropriate food *ad libitum*.

During the study, the well-being and tolerability of the rats were continuously assessed via cage-side observation. Parameters documented included behavioral changes, wounds, hemorrhage, and unusual events. Additionally, the Mouse Grimace Scale^16^, an objective measure based on facial expressions, was utilized to aid in the evaluation of potential pain.

Surgical procedures were performed following antisepsis and asepsis protocols, and the animals were anesthetized with a solution of 0.5 mL of 2% xylazine hydrochloride, 1 mL of 10% ketamine hydrochloride, 0.6 mL of 0.5% midazolam maleate, and 8.5 mL of 0.9% saline solution. One milliliter per 100 g of animal body weight of this solution was delivered, via intraperitoneal route.

After confirming the correct anesthetic plan, each rat was placed in dorsal decubitus with all four limbs extended. The anal sphincter was carefully dilated using a blunt and delicate dissection forceps. A number 5 surgical steel wire with a ½-turn cutting needle of 48 mm in length was passed from the rectum to approximately 1 cm from the anal verge. This wire was cut and twisted to form a wide loop at the site, similar to a loose seton. For each animal, two steel wires were placed in diametrically opposite positions, specifically, at 3 and 9 o’clock. The animals were monitored until they had fully recovered from anesthesia and then were kept in collective cages, and given free access to water and food. In 10 animals, the loose seton was created in a simplified manner: instead of twisting the steel wire, the two cut ends were folded and joined.

Thirty days after the placement of the metal setons, all the animals were anesthetized again, and the steel wires were removed.

Subsequently, two equal groups of 13 rats were established. In the first group, tissue specimens were harvested for histopathological examination two weeks after seton thread removal. In the second group, tissue specimens were harvested four weeks after seton thread removal to compare the changes involved in anal fistula formation at these two distinct time points. All animals were euthanized via intracardiac injection of thiopental under anesthesia.

After local trichotomy, the external fistula orifices, both on the left and right sides, were identified and included in the *en bloc* resection of the perianal region and lower rectum. Each sample was then divided in half, and each half was identified and prepared for histopathological analysis. In this way, each resulting specimen had a cutaneous border corresponding to the perianal region and a mucosal border including the lower rectum.

The tissue that was used for histopathological study was kept open, using a small Styrofoam plate and four pins, to preserve its spatial orientation and avoid loss of the anatomical reference. This tissue was preserved for 48 hours in a 10% buffered formalin solution, maintained for another 72 hours in a 70% alcoholic solution and then cleaved.

During the cleavage process, the longitudinal cut in the tissue followed the skin/mucosa orientation; that is, it started at the cutaneous edge of the tissue and ended at its mucosal edge. When the external opening of the fistula could be identified, the cut was made following this skin/mucosa axis and over the external opening with the intention of dividing the possible fistulous tract in half in the longitudinal direction. In tissue pieces in which identification of the external opening was not possible or in tissue pieces in which this was unclear, the cut was made on the same skin/mucosa axis to divide the piece into two halves. When the halves obtained with this first cut were wider than 5 mm, they were cut again in the same longitudinal direction. Thus, from each of the two original anorectal segments (right and left), two to four fragments were obtained for the preparation of the histological slides. Each fragment of the piece was then placed in a small fold of filter paper, with a mark on the side to be initially cut to obtain the slides to be studied.

These slides, which were stained with hematoxylin and eosin, were analyzed via optical microscopy, to assess the presence of the fistula tract, degree of healing, presence of fibrosis, and the cellularity and inflammatory pattern observed, in addition to the presence or absence of epithelial cells in the fistula tract. Any other observed characteristics were also recorded.

## Results

The total of 26 male Wistar rats underwent surgery in two distinct phases. The initial phase, involving six rats, served as a pilot study to clarify doubts and refine the surgical process. Following this pilot phase, the remaining 20 rats were operated on in the second phase. Two fistulas were produced in each animal, resulting in a total of 52 fistulas. The average weight of the animals at the beginning of the experiment was 311 grams, and at the end of the first 30 days of the experiment, an average of 376 grams was weighed. An average weight gain of 64.3 grams was observed after thirty days.

Daily monitoring of all animals in their cages throughout the experiment indicated absence of suffering. Specifically, mouse grimace scale scores were consistently 0 (not present) for all assessed facial action units (orbital tightening, nose bulge, cheek bulge, ear position, and whisker change). Moreover, no behavioral changes, wounds, or bleeding were noted, even in the four rats that subsequently developed abscesses or those that lost their seton.

At the end of the experiment, eight setons were lost, leaving a total of 44 fistulas to be studied. Only one rat lost both setons and was excluded from the study, resulting in a 96% utilization rate of the animals. There were no animal deaths.

All 44 remaining fistulas could be identified by observing the scar of the external orifice two and four weeks after the removal of the metal wire. Four fistulas (4/44) presented external signs of local infection, indicated by the formation of non-drained abscesses, one in the two-week group (1/22) and three in the four-week group (3/22). Five days after the removal of the anatomical specimen for the anatomopathological study, in the cleavage phase of the material, the external orifice of the fistula could still be identified in 28 specimens (64%). Among them, 16 specimens belonged to the two-week group (16/22) and 12 specimens belonged to the four-week group (12/22). The findings did not differ significantly between the two-week and four-week groups.

## Discussion

Several experimental models have been proposed for research on anal fistulas. Some of these models use larger animals, such as pigs, weighing between 38 and 41 kg^15^. Working with larger animals allows the anatomy of the animal model to more closely resemble humans’ one and facilitates the evaluation of fistulas form via magnetic resonance imaging, which is the gold standard method for this type of evaluation. However, the logistics required to handle and maintain animals such as these pigs may make studies with a larger number of animals or studies with a lower budget unfeasible. The use of rats for this purpose has several benefits, such as ease of breeding and handling, low financial cost, and fast animal metabolism, which shorten the study time, as well as their great regenerative capacity and resistance to adversity. For this reason, rats are widely used as models^6^–^9^.

The approach of inducing two fistulas in a single animal has some strategic, economic, and ethical advantages. In this type of approach, a single animal can provide homogeneous information for both the control group and the study group in an experimental model. Therefore, if the same animal is included in both the control and study groups, both groups will experience equivalent systemic stress due to the experimental procedure. This minimizes intergroup variability, increases homogeneity and aligns with the 3Rs principle of animal research: replacement, reduction, and refinement^17^,^18^.

The model studied proved to be quite viable in this regard, given that it was well tolerated by the animals. During daily observation of the rats, no signs of stress or pain due to the presence of the metal seton were observed between the phases of the experiment. The general behavior of the rats in the cages was observed, and their facial expression was also analyzed according to the mouse grimace scale^16^. As a result, weight gain was also observed throughout the procedure. The median weight gain/day was 2.16 grams, ranging from 0.2 to 3.66 grams. This observation also resulted in the possibility of the animals being housed in collective cages without causing harm to any weaker animals. This translates into an advantage for the well-being of gregarious animals, such as rats.

Various materials have been used experimentally to induce anal fistulas. Some desirable characteristics of these materials include ease of handling, accessibility, and, if possible, a local reaction that leads to the formation of a fistulous tract that resembles the one found naturally. As previously described, epithelialization of the fistulous tract plays a key role in the formation and maintenance of anal fistulas^19^–^21^. Therefore, the material that is used to experimentally induce fistulas must be capable of stimulating complete or partial epithelialization of the created tract. Another factor to be considered is fistulous tract contamination. In fistulas of cryptoglandular origin, it has been suggested that contamination of the fistulous tract occurs at the expense of the microbiota of intestinal origin^22^. Therefore, the material that is used to experimentally induce a fistula must not harm the microorganisms that are introduced in this hole, produce large entry and exit holes^23^, and the direction of material introduction–from the rectum to the skin–must be designed in such a way to reproduce the contamination that occurs naturally in fistulas of cryptoglandular origin. The most commonly used materials for the experimentally induction of anal fistulas are rubber strips^10^,^24^, nylon^15^, polyglactin thread (Vicryl)^23^, silicone tubes (Silastic)^15^,^24^, polyvinyl chloride-coated aluminum wires^9^, and, most commonly, steel wires^6^,^8^. Surgical steel wire offers several advantages as a seton material. Firstly, steel elicits an inflammatory response, crucial for inducing a fistula with a fully or partially epithelialized tract^6^. Secondly, its hardness ensures resistance to damage from the animal’s teeth. Thirdly, steel exhibits excellent biocompatibility, with minimal corrosion during the experimental period. Finally, owing to the cut produced by the needle, it forms large entry and exit holes. Another important consideration is that the procedure for inducing the fistula must be performed in a manner that does not cause pain, and the fistula should remain pain-free. In this way, there will be little stimulus for the animal to try to remove the seton.

**Figure 1:**
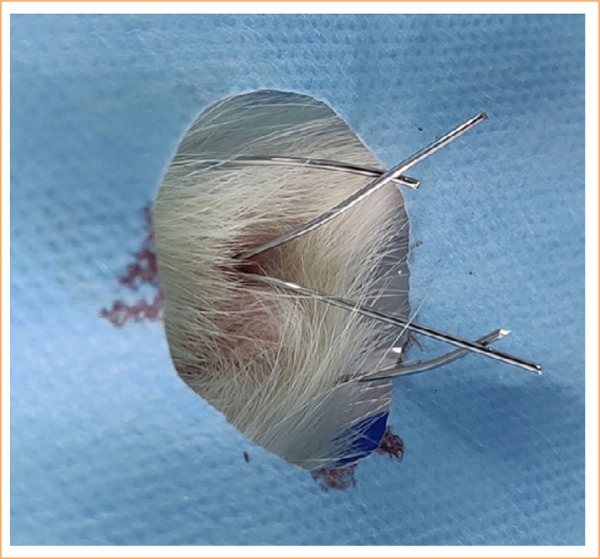
Untwisted seton.

Source: Elaborated by the authors.

In the present study, eight metal setons were lost. Only one animal lost both setons and had to be excluded from the study. The other six affected animals lost only one of the setons and could be included in the experiment, thus minimizing losses. The loss of the seton can be explained by a variation of the technique applied in a portion of 10 animals operated on in the second phase of the study. In the original method proposed, each metal wire made it possible to generate up to six setons. Twisting and removing the wire can be problematic if an adequate cutting tool is not available. For this reason, a technique that does not twist the wire completely but only folds it, maintaining contact between its ends without twisting, was attempted (Fig. 1). However, the interactive ability of the rats appears to have been underestimated. None of the seven rats that lost the seton showed signs of bleeding in the cage or injuries/scars caused by the avulsion of the wire. Thus, it was concluded that the animals were able to manage objects and, therefore, were able to remove the seton.

**Figure 2:**
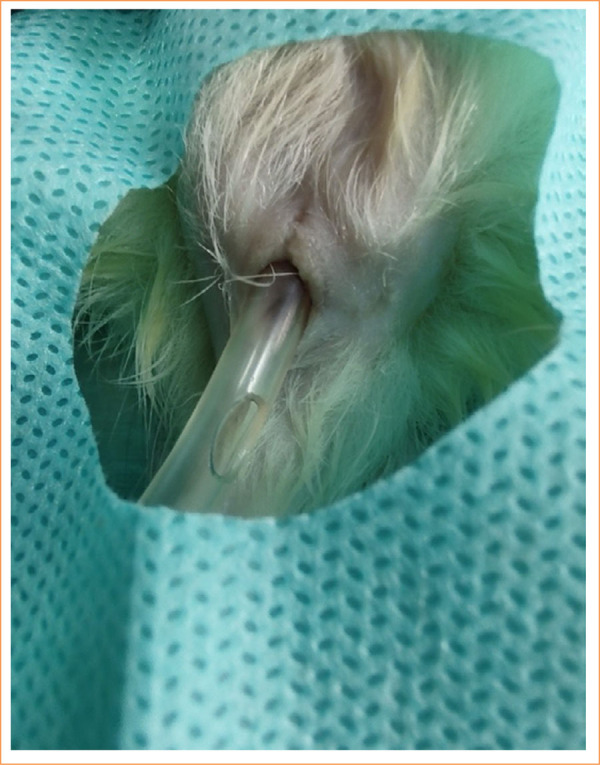
Stabilization of the rectum for *en bloc* resection of anatomical specimen, perianal region, and lower rectum.

Source: Elaborated by the authors.

The collection of material for anatomopathological studies also requires some care, especially if the approach is performed exclusively via the perineal route. Rats have a significant excess of tissue in this anatomical region, which is highly mobile. This can present a challenge for good local dissection. It is common to have difficulty in precisely cutting with the scalpel, and if care is not taken, the most distal part of the fistulous tract may be left out. One way to make this step easier is to stabilize this region by introducing the blunt tip of a number 8 Fr rectal catheter and using scissors with a perfect cut. This facilitates *en bloc* resection of the specimen, keeping the rectum well aligned and facilitating the division of the specimen between its opposite halves (Fig. 2).

**Figure 3:**
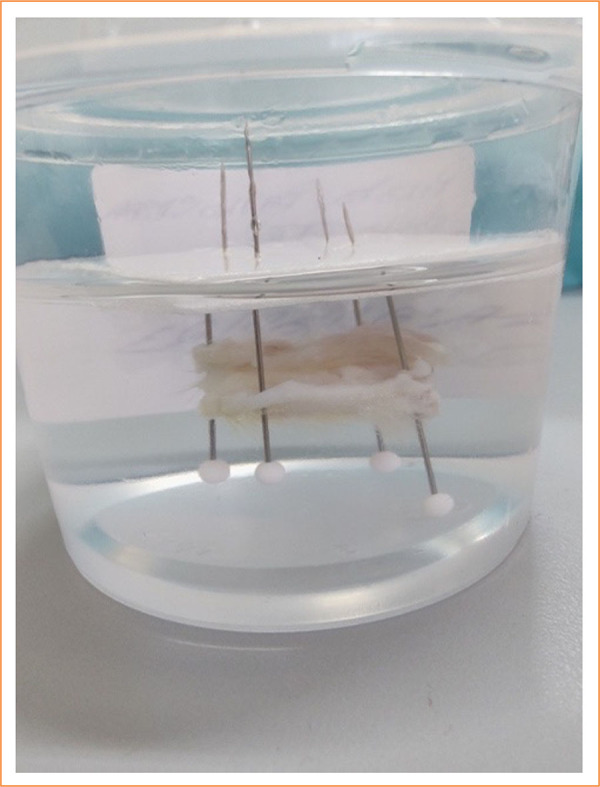
Piece fixed in 10% formalin solution.

Source: Elaborated by the authors.

**Figure 4:**
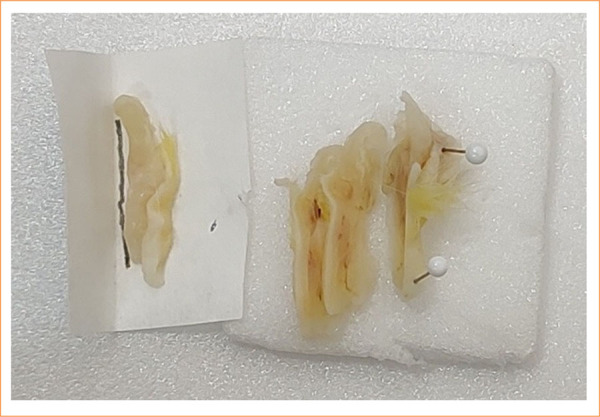
Material cleaved and (on the left) placed on marked filter paper.

Source: Elaborated by the author

Another important consideration is the cleavage stage of the sample used to obtain the tissues for histopathological study. Importantly, the specimen must be correctly cleaved, its orientation in the distal/proximal direction of the fistula must be maintained, and the correct side for making the cuts with the microtome must be identified, as this allows the maximum possible extension of the fistulous tract for histological evaluation. A first step should be taken when the sample is fixed in formalin. The removed sample still needs to be stretched and stabilized, as the dehydration caused by formalin leads to shrinkage and, often, twisting of the sample. This makes it difficult to identify the anatomical landmarks and to achieve good cleavage. A useful resource that is used to stabilize a sample is to fix it with four pins on a small plate. Importantly, full contact of the entire removed sample with the formalin fixative solution was maintained (Fig. 3). If the researcher is not the same one who will prepare the histological slide or if s/he will not do so immediately, it may be useful to accommodate the cleaved pieces in small folds of filter paper, with the appropriate marking of the side to be cut by the microtome and, in this way, ensure good material for microscopic study (Fig. 4).

The experiment was conducted to investigate the dynamics of anal fistula formation. To achieve a deeper understanding of this process, the evaluation was performed at two distinct and well-defined time points. This longitudinal approach allowed for the analysis of morphological and inflammatory changes occurring over time, enabling us to assess whether these alterations persist. In our observation, the changes remained relatively consistent in both the two- and four-week groups. Thus, we can suggest that other experiments, based on our model, could be performed over shorter or longer durations depending on the specific objective of each new experiment. This would be an advantage of the experimental model studied. However, the histopathological examination of an event interrupts its continuity, which may introduce bias regarding its outcome. Combined alternatives of histopathological examination and magnetic resonance imaging could address this gap.

Complete evaluation of the fistula area (proximal, middle, and distal segments) is facilitated by the longitudinal sectioning of the specimen. However, the fistula tract is not always linear and may not be fully present on a single histological slide, requiring additional serial sections for a detailed histopathological analysis. A transverse section is an alternative, presenting a cross-section of the fistula tract and reducing the number of slides needed, but this may hinder a detailed assessment of the fistula’s total extent.

## Conclusion

The experimental model studied here proved to be viable, reproducible, and low cost. Its economic and time efficiency allows for its use in a series of studies investigating the treatment of anal fistulas.

## Data Availability

The datasets used and analyzed during the current study are available from the corresponding author on reasonable request.
